# Safety of Re-dosing Nirsevimab Prior to RSV Season 2 in Children With Heart or Lung Disease

**DOI:** 10.1093/jpids/piad052

**Published:** 2023-07-19

**Authors:** Joseph B Domachowske, Yue Chang, Victoria Atanasova, Fernando Cabañas, Kenji Furuno, Kim A Nguyen, Irfana Banu, Robert J Kubiak, Amanda Leach, Vaishali S Mankad, Manish Shroff, Therese Takas, Tonya Villafana, Ulrika Wählby Hamrén

**Affiliations:** Department of Pediatrics, SUNY Upstate Medical University, Syracuse, New York, USA; Vaccines & Immune Therapies, Biopharmaceuticals R&D, AstraZeneca, Gaithersburg, Maryland, USA; UMHAT Dr. G. Stranski EAD, Clinic of Neonatology, Pleven, Bulgaria; Department of Paediatrics-Neonatology, Quironsalud Madrid University Hospital and Quironsalud San José Hospital, Madrid, Spain; Department of General Pediatrics and Interdisciplinary Medicine, Fukuoka Children’s Hospital, Fukuoka, Japan; Hospices Civils de Lyon, Hôpital Femme-Mere-Enfant, Service de Réanimation Néonatale et de Neonatologie (NICU) et CIC 1407, Lyon, France; Vaccines & Immune Therapies, BioPharmaceuticals R&D, AstraZeneca, Cambridge, UK; Clinical Pharmacology and Quantitative Pharmacology, BioPharmaceuticals R&D, AstraZeneca, Gaithersburg, Maryland, USA; Vaccines & Immune Therapies, Biopharmaceuticals R&D, AstraZeneca, Gaithersburg, Maryland, USA; Vaccines & Immune Therapies, BioPharmaceuticals R&D, AstraZeneca, Durham, North Carolina, USA; Patient Safety, Chief Medical Office, R&D, AstraZeneca, Waltham, Massachusetts, USA; Vaccines & Immune Therapies, Biopharmaceuticals R&D, AstraZeneca, Gaithersburg, Maryland, USA; Vaccines & Immune Therapies, Biopharmaceuticals R&D, AstraZeneca, Gaithersburg, Maryland, USA; Clinical Pharmacology and Quantitative Pharmacology, R&D, AstraZeneca, Gothenburg, Sweden

**Keywords:** monoclonal antibody, nirsevimab, pharmacokinetics, palivizumab, respiratory syncytial virus

## Abstract

In children with congenital heart disease and/or chronic lung disease entering their second respiratory syncytial virus (RSV) season, 200 mg nirsevimab had a similar safety profile to that of palivizumab and resulted in nirsevimab serum exposures associated with efficacy in healthy infants, supporting efficacy in this population at risk of severe RSV disease.

Nirsevimab, a monoclonal antibody with an extended half-life, has been developed to protect infants against lower respiratory tract infection due to respiratory syncytial virus (RSV) [1, 2]. Following a single dose of nirsevimab before the first RSV season, efficacy was demonstrated and safety described in otherwise healthy preterm infants in the Phase 2b (≥29 to <35 weeks gestational age [GA]) and Phase 3 MELODY (≥35 weeks GA) trials [3–5]. Extrapolation of efficacy based on pharmacokinetics (PK) is an established means of extending label claims to other populations [6]. Based on the mechanism of action of nirsevimab, efficacy was extrapolated to infants with congenital heart disease (CHD), chronic lung disease of prematurity (CLD), and those born extremely preterm [7] in the Phase 2/3 MEDLEY trial (NCT03959488) [8]. Furthermore, the nirsevimab safety profile was similar to that of palivizumab during the first RSV season in MEDLEY [8]. Here we report the safety and PK extrapolation of efficacy among children with CHD or CLD following administration of a second nirsevimab dose prior to their second RSV season.

## METHODS

The methodology of the MEDLEY trial has been described previously [8]. In brief, infants were required to be eligible to receive palivizumab in accordance with local or national guidelines; eligibility for participation in a second season was restricted to infants with CHD or CLD in accordance with these guidelines. Before their first RSV season, participants were randomly assigned to receive nirsevimab in a single intramuscular dose of 50 mg (if weight <5 kg) or 100 mg (if weight ≥5 kg), followed by four once-monthly doses of placebo, or five once-monthly intramuscular doses of palivizumab (15 mg/kg weight per dose) (Supplementary Figure 1). Prior to the second season, participants with CHD or CLD randomized to nirsevimab before the first season received 200 mg nirsevimab followed by four once-monthly doses of placebo (N/N); those randomized to palivizumab were re-randomized 1:1 to either 200 mg nirsevimab followed by four once-monthly doses of placebo (P/N) or five once-monthly intramuscular doses of palivizumab (15 mg/kg weight per dose) (P/P). Adverse events (AEs), antidrug antibodies (ADA), and nirsevimab serum concentrations were assessed through 360 days post first Season 2 dose, representing the active nirsevimab dose for the P/N and N/N groups and the first of five active palivizumab doses for the P/P group. PK was evaluated, and area under the curve data were derived, as previously described [7]. The success criterion for PK extrapolation was 80% of participants achieving the previously defined nirsevimab exposure target associated with efficacy (12.8 day·mg/mL) [7]. AEs of special interest (Type I hypersensitivity [including anaphylaxis], immune complex disease, and thrombocytopenia) were assessed by investigators. Data from an interim analysis are presented, including safety, ADA, and PK evaluation ≥150 days post first Season 2 dose (corresponding to a typical 5-month RSV season). Data for this analysis were collected from July 28, 2020–April 30, 2022. The safety analysis included an assessment of AEs through 30 days post first dose to permit an evaluation of AEs relative to receiving nirsevimab compared with one palivizumab dose.

## RESULTS

Of 310 participants with CHD/CLD enrolled prior to the first season, 262 continued to the second season (42 P/P, 40 P/N, 180 N/N) and 252 completed ≥150 days follow-up, post first Season 2 dose (Supplementary Figure 2). The characteristics of children continuing to Season 2 were similar between treatment groups (Table 1). The incidence of AEs during the second season was similar across treatment groups (P/P: 29 [69.0%]; P/N: 29 [72.5%]; N/N: 126 [70.0%]), with no deaths or AEs of special interest reported (Table 2). The overall incidence of serious AEs and AEs of Grade 3 or greater severity was numerically higher among P/N and N/N recipients than P/P recipients (Table 2); these events were primarily due to infections or were related to underlying comorbid conditions and no trends or safety concerns were identified (Supplementary Tables 1 and 2). Within 30 days post first Season 2 dose, the incidence of serious AEs and AEs of Grade 3 or greater severity was similar among the three treatment groups (Table 2). The incidence of AEs post first Season 2 dose by time relative to any dose was also similar (Supplementary Tables 3 and 4).

**Table 1.** Characteristics of Children With CHD/CLD Continuing to RSV Season 2 (ITT Population)

Characteristic	P/P, *n* (%) (*N *= 42)	P/N^a^, *n* (%) (*N *= 40)	N/N, *n* (%) (*N* = 180)	Total^a^, *n* (%) (*N* = 262)
Gestational age group, *n* (%)				
<29 weeks	16 (38.1)	16 (40.0)	71 (39.4)	103 (39.3)
≥29 to <32 weeks	8 (19.0)	2 (5.0)	33 (18.3)	43 (16.4)
≥32 to <35 weeks	7 (16.7)	4 (10.0)	25 (13.9)	36 (13.7)
≥35 weeks	11 (26.2)	18 (45.0)	51 (28.3)	80 (30.5)
Age at start of Season 2, median months (range)	15.8 (12.5–19.9)	16.4 (12.5–22.3)	16.7 (12.1–23.2)	16.6 (12.1–23.2)
Weight at start of Season 2, median kg (range)	9.9 (6.3–12.7)	9.8 (6.4–14.9)	9.7 (6.1–15.7)	9.8 (6.1–15.7)
Existing heart or lung disease, *n* (%)				
CHD^b^	11 (26.2)	14 (35.0)	56 (31.1)	81 (30.9)
VSD	1 (9.1)	4 (28.6)	18 (32.1)	23 (28.4)
Atrial septal defect	2 (18.2)	2 (14.3)	6 (10.7)	10 (12.3)
Tetralogy of Fallot	3 (27.3)	0	5 (8.9)	8 (9.9)
Single ventricle including hypoplastic left or right heart	0	2 (14.3)	5 (8.9)	7 (8.6)
Atrioventricular septal defect	1 (9.1)	2 (14.3)	4 (7.1)	7 (8.6)
dextro-TGA with or without VSD and with or without pulmonary stenosis	0	2 (14.3)	2 (3.6)	4 (4.9)
Patent foramen ovale	0	0	3 (5.4)	3 (3.7)
Pulmonary atresia with VSD	1 (9.1)	1 (7.1)	1 (1.8)	3 (3.7)
Tricuspid atresia	1 (9.1)	0	1 (1.8)	2 (2.5)
Abnormal lung vein drainage, restrictive PDA	0	0	1 (1.8)	1 (1.2)
Aortic stenosis post valvuloplasty	0	0	1 (1.8)	1 (1.2)
Aortic valve stenosis	0	0	1 (1.8)	1 (1.2)
Coarctation of the aorta complex	0	0	1 (1.8)	1 (1.2)
Double outlet right ventricle with TGA	0	0	1 (1.8)	1 (1.2)
Hemodynamically significant residual coarctation of the aorta after anastomosis	0	0	1 (1.8)	1 (1.2)
Interrupted aortic arch Type B, VSD, bicuspid aortic valve, status post ductus arteriosus stenting with LPA and RPA banding	0	0	1 (1.8)	1 (1.2)
Pulmonary arterial stenosis	0	0	1 (1.8)	1 (1.2)
Pulmonary atresia with intact septum	0	0	1 (1.8)	1 (1.2)
Pulmonary atresia with intact ventricular septum	0	0	1 (1.8)	1 (1.2)
VSD and aortic coarctation	0	0	1 (1.8)	1 (1.2)
Interrupted aortic arch Type B and VSD	0	1 (7.1)	0	1 (1.2)
Aortic stenosis	1 (9.1)	0	0	1 (1.2)
Ebstein’s anomaly	1 (9.1)	0	0	1 (1.2)
CLD^b^	32 (76.2)	25 (62.5)	132 (73.3)	189 (72.1)

Prior to the second season, children with CHD/CLD randomized to nirsevimab in the first season received 200 mg nirsevimab followed by four once-monthly doses of placebo (N/N) and those randomized to palivizumab in the first season were re-randomized 1:1 to either 200 mg nirsevimab followed by four once-monthly doses of placebo (P/N) or five once-monthly intramuscular doses of palivizumab (15 mg/kg of body weight per dose) (P/P). The primary cardiac lesion as reported by the investigator is provided for participants with CHD; percentage of participants with specific lesions are calculated using the total number of participants with CHD as the denominator.

Abbreviations: CHD, congenital heart disease; CLD, chronic lung disease of prematurity; ITT, intent-to-treat; LPA, left pulmonary artery; N, nirsevimab; P, palivizumab; PDA, patent ductus arteriosus; RPA, right pulmonary artery; TGA, transposition of the great arteries; VSD, ventricular septal defect.

^a^Includes one participant with Down syndrome without CLD or CHD.

^b^Participants with both CHD and CLD (*n* = 9; 1 in the P/P group, 8 in the N/N group) are counted in both the CLD and CHD subpopulations.

**Table 2.** Overall Summary of Adverse Events Through ≥150 Days Post First Dose^a^ of RSV Season 2 (AT population)

Adverse events^b^	P/P, *n* (%) (*N *= 42)	P/N, *n* (%) (*N* = 40)	N/N, *n* (%) (*N *= 180)	Total, *n* (%) (*N* = 262)
1 or more	29 (69.0)	29 (72.5)	126 (70.0)	184 (70.2)
Occurring ≤1 day post first dose^a^	0 (0.0)	0 (0.0)	2 (1.1)	2 (0.8)
Occurring ≤3 days post first dose^a^	0 (0.0)	1 (2.5)	4 (2.2)	5 (1.9)
Occurring ≤7 days post first dose^a^	2 (4.8)	4 (10.0)	7 (3.9)	13 (5.0)
Occurring ≤14 days post first dose^a^	9 (21.4)	4 (10.0)	28 (15.6)	41 (15.6)
Occurring ≤30 days post first dose^a^	11 (26.2)	11 (27.5)	54 (30.0)	76 (29.0)
One or more treatment-related	0 (0.0)	0 (0.0)	0 (0.0)	0 (0.0)
One or more Grade 3 or greater severity^c^	1 (2.4)	4 (10.0)	14 (7.8)	19 (7.3)
Occurring ≤1 day post first dose^a^	0 (0.0)	0 (0.0)	0 (0.0)	0 (0.0)
Occurring ≤3 days post first dose^a^	0 (0.0)	0 (0.0)	0 (0.0)	0 (0.0)
Occurring ≤7 days post first dose^a^	0 (0.0)	0 (0.0)	0 (0.0)	0 (0.0)
Occurring ≤14 days post first dose^a^	0 (0.0)	0 (0.0)	0 (0.0)	0 (0.0)
Occurring ≤30 days post first dose^a^	1 (2.4)	1 (2.5)	3 (1.7)	5 (1.9)
One or more serious^d^	0 (0.0)	4 (10.0)	17 (9.4)	21 (8.0)
Occurring ≤30 days post first dose^a^	0 (0.0)	1 (2.5)	4 (2.2)	5 (1.9)
One or more with outcome of death	0 (0.0)	0 (0.0)	0 (0.0)	0 (0.0)
One or more of special interest^e^	0 (0.0)	0 (0.0)	0 (0.0)	0 (0.0)
One or more related to COVID-19^f^	5 (11.9)	4 (10.0)	13 (7.2)	22 (8.4)
Occurring ≤30 days post dose^a^	0 (0.0)	1 (2.5)	3 (1.7)	4 (1.5)

Prior to the second season, children with CHD/CLD randomized to nirsevimab in the first season received 200 mg nirsevimab followed by four once-monthly doses of placebo (N/N) and those randomized to palivizumab in the first season were re-randomized 1:1 to either 200 mg nirsevimab followed by four once-monthly doses of placebo (P/N) or five once-monthly intramuscular doses of palivizumab (15 mg per kilogram of body weight per dose) (P/P). Adverse events were coded by MedDRA Version 23.1 or higher, unless otherwise stated. There were no treatment-related serious adverse events or serious adverse events of Grade 3 or greater severity.

Abbreviations: AT, as treated; COVID-19, coronavirus disease 2019; CHD, congenital heart disease; CLD, chronic lung disease; MedDRA, Medical Dictionary for Regulatory Activities; N, nirsevimab; P, palivizumab.

^a^Relative to the active nirsevimab dose for P/N and N/N groups and to the first of 5 active doses for the P/P group.

^b^Participants with multiple events in the same category were counted once in that category; participants with events in >1 category were counted once in each of those categories.

^c^An adverse event of Grade 3 denotes a severe event, an adverse event of Grade 4 a life-threatening event, and an adverse event of Grade 5 a fatal event.

^d^Serious adverse event criteria: death, life-threatening, required inpatient hospitalization, prolongation of existing hospitalization, persistent or significant disability/incapacity, important medical event, congenital anomaly/birth defect.

^e^Includes Type I hypersensitivity (including anaphylaxis), immune complex disease, and thrombocytopenia.

^f^COVID-19–related adverse events were symptomatic or asymptomatic COVID-19 cases with a confirmatory diagnostic test positive for severe acute respiratory syndrome coronavirus 2 or suspected cases for which signs and symptoms were judged by the investigator to be highly suspicious for COVID-19 but for which a confirmatory diagnostic test was unavailable or negative.

The ADA incidence was low in participants who received N/N, occurring in 1/90 participants (1.1%) at Day 31 and in 0/158 participants at Day 151; no participant with post baseline ADA in the first season had detectable ADA in the second season. The PK exposure target for efficacy extrapolation was met, with 98% of participants achieving target serum AUCs across the P/N and N/N treatment groups. No RSV lower respiratory tract infections occurred through Day 151.

## DISCUSSION

In this study of children with CLD/CHD entering their second RSV season, the safety profile observed was favorable for nirsevimab and similar to that of palivizumab. Additionally, a second dose of nirsevimab was associated with a low incidence of ADA, and no Type I hypersensitivity was observed with re-exposure, including among participants who were ADA positive at the end of Season 1.

Participants with CHD/CLD who received a 200 mg dose of nirsevimab before their second RSV season achieved nirsevimab serum exposures known to be efficacious in preventing MA RSV LRTI in healthy term and preterm infants. Efficacy is, therefore, expected for children with CHD/CLD entering their second season.

One limitation is that the re-randomization of the palivizumab recipients in the first season meant that the P/P and P/N groups were relatively small. In addition, the trial enrolled and collected safety data during the coronavirus disease 2019 pandemic, which is known to have affected RSV circulation. Given the unpredictable situation in the face of the pandemic and the primary objective of the study being safety, it was decided to continue with the study as planned using historical conventions of seasonality in the respective regions where infants were enrolled (all but 4 of the 262 participants participating in the second season were enrolled in the Northern Hemisphere), with the season defined as lasting 150 days (corresponding to a typical 5-month RSV season). Approximately half of the participants went through their second RSV season during 2020, when the COVID-19 pandemic impacted RSV epidemiology. The other half of the participants went through their second RSV season during 2021, when transmission was occurring but with a somewhat atypical seasonality. Logistical challenges presented by the pandemic were addressed by ensuring regular contact with participants via phone and telemedicine. Of note, there were no cases of medically attended RSV LRTI through 150 days post first Season 2 dose during MEDLEY. Aside from the impact of the pandemic on RSV circulation, other potential reasons include the age of participants, the inclusion of an active comparator, and the relatively small number of children participating in the second season of the study (*n* = 262).

In conclusion, these findings demonstrate a favorable safety profile for nirsevimab in children with CHD/CLD entering their second RSV season, with PK data supporting efficacy in this population of children who are among those at the highest risk of severe RSV disease.

## Supplementary Material

piad052_suppl_Supplementary_Material

## Data Availability

Data underlying the findings described in this manuscript may be obtained in accordance with AstraZeneca’s data sharing policy described at https://astrazenecagrouptrials.pharmacm.com/ST/Submission/Disclosure. Data for studies directly listed on Vivli can be requested through Vivli at www.vivli.org. Data for studies not listed on Vivli could be requested through Vivli at https://vivli.org/members/enquiries-about-studies-not-listed-on-the-vivli-platform/. AstraZeneca Vivli member page is also available outlining further details: https://vivli.org/ourmember/astrazeneca/.

## Notes

***Financial support:*** This study was supported by AstraZeneca and Sanofi. AstraZeneca was involved in the trial design; the collection, analysis, and interpretation of the data; and the writing of the manuscript. Nirsevimab is being developed and commercialized in partnership between AstraZeneca and Sanofi.

***Potential conflicts of interest.*** J. B. D. has received consulting fees from Sanofi; payment or honoraria from Sanofi; and has participated in data safety monitoring boards or advisory boards for AstraZeneca. V. A. and K. A. N. report no conflicts of interest. K. F. has received payment or honoraria from AstraZeneca. F. C. is a member of the Paediatric Committee at the European Medicines Agency (EMA) but has not participated in the deliberations or decisions related to this product (as communicated to the EMA). Y. C., I. B., R. J. K., A. L., V. S. M., M. S., T. T., T. V., and U. W. H. are employees of AstraZeneca and may hold stock or stock options.

## Clinical Trial Registration

Clinicaltrials.gov, NCT03959488, https://clinicaltrials.gov/ct2/show/NCT03959488.

## References

[1] Griffin MP , Khan AA, Esser MT, et al. Safety, tolerability, and pharmacokinetics of MEDI8897, the respiratory syncytial virus prefusion F-targeting monoclonal antibody with an extended half-life, in healthy adults. Antimicrob Agents Chemother 2017; 61:e01714–16. 27956428 10.1128/AAC.01714-16 PMC5328523

[2] Li Y , Wang X, Blau DM, et al.; Respiratory Virus Global Epidemiology Network. Global, regional, and national disease burden estimates of acute lower respiratory infections due to respiratory syncytial virus in children younger than 5 years in 2019: a systematic analysis. Lancet 2022; 399:2047–64. 35598608 10.1016/S0140-6736(22)00478-0 PMC7613574

[3] Griffin MP , Yuan Y, Takas T, et al.; Nirsevimab Study Group. Single-dose nirsevimab for prevention of RSV in preterm infants. N Engl J Med 2020; 383:415–25. 32726528 10.1056/NEJMoa1913556

[4] Hammitt LL , Dagan R, Yuan Y, et al.; MELODY Study Group. Nirsevimab for prevention of RSV in healthy late-preterm and term infants. N Engl J Med 2022; 386:837–46. 35235726 10.1056/NEJMoa2110275

[5] Muller WJ , Madhi SA, Seoane Nuñez B, et al.; MELODY Study Group. Nirsevimab for prevention of RSV in term and late-preterm infants. N Engl J Med 2023; 388:1533–4. 37018470 10.1056/NEJMc2214773

[6] Mulugeta Y , Barrett JS, Nelson R, et al. Exposure matching for extrapolation of efficacy in pediatric drug development. J Clin Pharmacol 2016; 56:1326–34. 27040726 10.1002/jcph.744 PMC5482171

[7] Simões EAF , Madhi SA, Muller WJ, et al. Efficacy of nirsevimab against respiratory syncytial virus lower respiratory tract infections in preterm and term infants, and pharmacokinetic extrapolation to infants with congenital heart disease and chronic lung disease: a pooled analysis of randomised controlled trials. Lancet Child Adolesc Health 2023; 7:180–9. 36634694 10.1016/S2352-4642(22)00321-2 PMC9940918

[8] Domachowske J , Madhi SA, Simões EAF, et al.; MEDLEY Study Group. Safety of nirsevimab for RSV in infants with heart or lung disease or prematurity. N Engl J Med 2022; 386:892–4. 35235733 10.1056/NEJMc2112186

