# Supplementary material for: Safety of Re-dosing Nirsevimab Prior to RSV Season 2 in Children With Heart or Lung Disease
Source: J Pediatric Infect Dis Soc. 2023 Jul 19;12(8):477–80. doi: 10.1093/jpids/piad052 (PMC10469583; doi:10.1093/jpids/piad052)
Supplement: piad052_suppl_Supplementary_Material [file piad052_suppl_supplementary_material.docx]

Supplement

**Supplemental Figure 1.** Study Design.


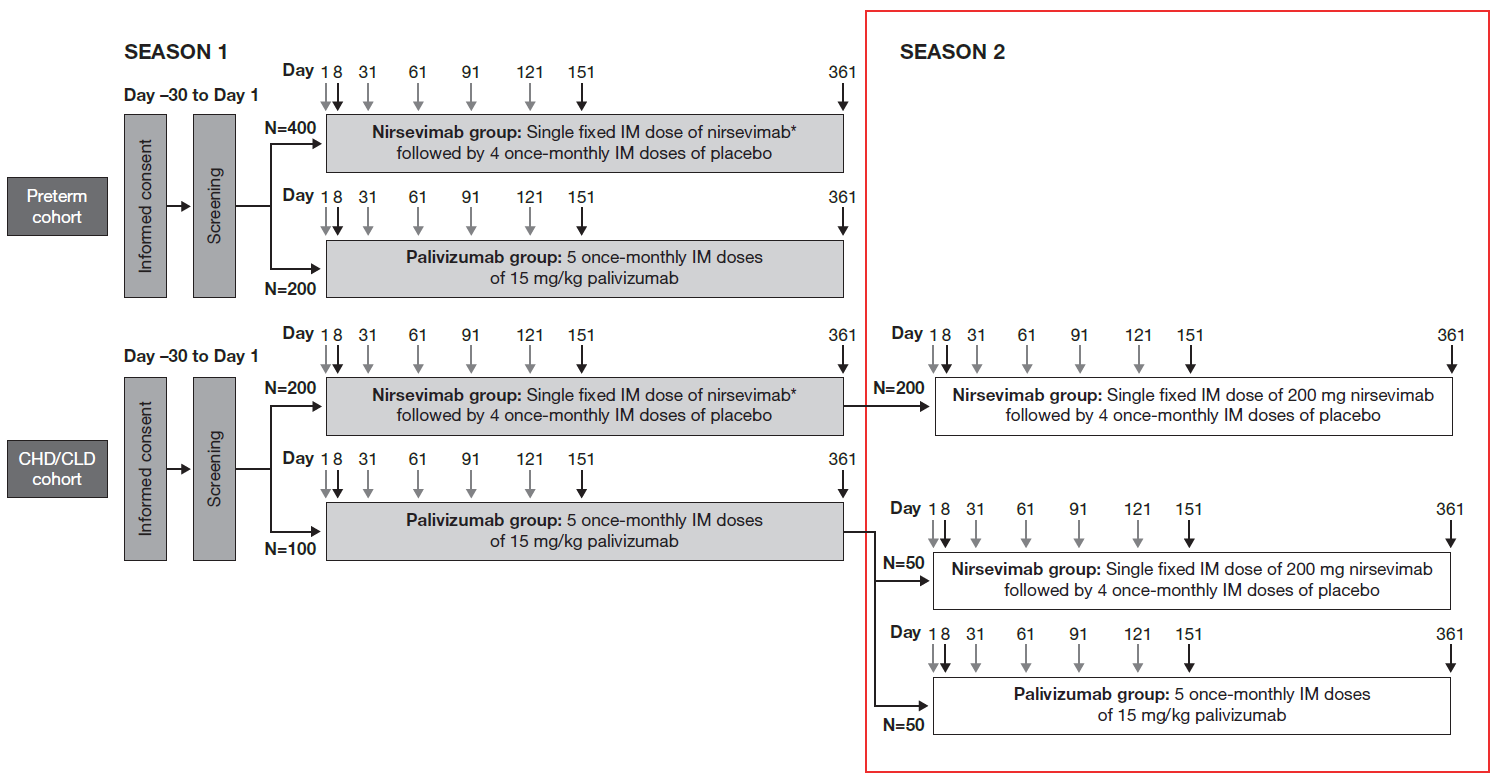


Gray arrows indicate once-monthly dosing of palivizumab (Day 1 only for nirsevimab); black arrows indicate follow-up visits.

*In the nirsevimab group Season 1, dose level was stratified by body weight at time of dosing; participants received nirsevimab 50 mg IM if <5 kg or 100 mg IM if ≥5 kg.

CHD, congenital heart disease; CLD, chronic lung disease of prematurity; IM, intramuscular.

**Supplemental Figure 2.** CONSORT diagram.


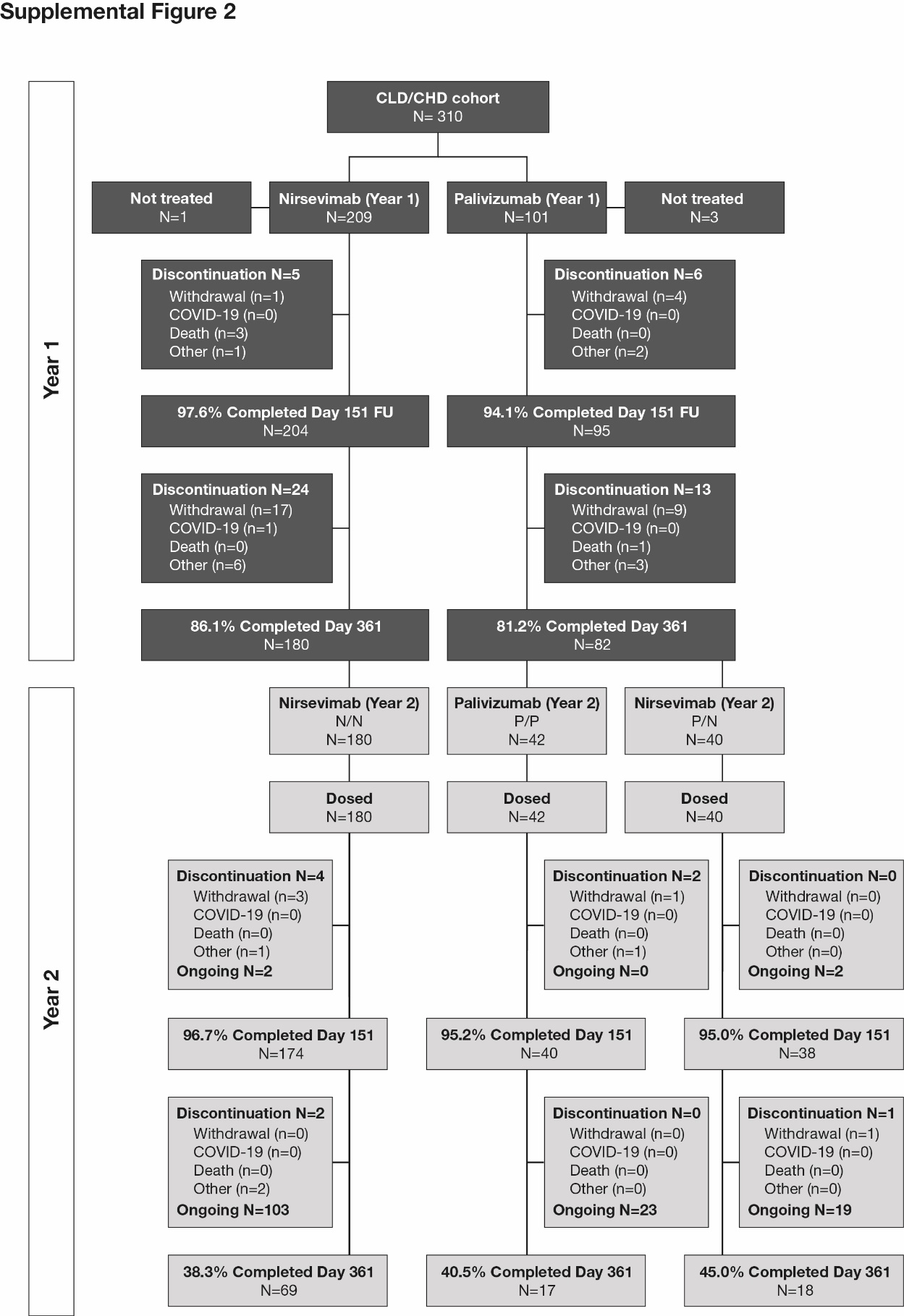


CHD, congenital heart disease; CLD, chronic lung disease of prematurity; COVID-19, coronavirus disease 2019; FU, follow-up; N, nirsevimab; P, palivizumab.

**Supplemental Table 1.** Treatment-Emergent Adverse Events of Grade 3 or Greater Severity through ≥150 Days Post First Dose^a^ of RSV Season 2 (AT population)

| Adverse events of Grade 3 or greater severity | Highest severity grade^b^ | P/P, *n* (%)  (*N* = 42) | P/N, *n* (%)  (*N* = 40) | N/N, *n* (%)  (*N* = 180) |
| --- | --- | --- | --- | --- |
| 1 or more | 3 | 1 (2.4) | 4 (10.0) | 13 (7.2) |
|  | 4 | 0 | 0 | 1 (0.6) |
| **Cardiac disorders** | **3** | **0** | **0** | **2 (1.1)** |
| Arrhythmia | 3 | 0 | 0 | 1 (0.6) |
| Atrioventricular block | 3 | 0 | 0 | 1 (0.6) |
| Cardiac failure | 3 | 0 | 0 | 1 (0.6) |
| **Congenital, familial and genetic disorders** | **4** | **0** | **0** | **1 (0.6)** |
| Fallot’s tetralogy | 4 | 0 | 0 | 1 (0.6)^c^ |
| **Gastrointestinal disorders** | **3** | **0** | **0** | **2 (1.1)** |
| Duodenal ulcer | 3 | 0 | 0 | 1 (0.6) |
| Intestinal obstruction | 3 | 0 | 0 | 1 (0.6) |
| **General disorders and administration site conditions** | **3** | **0** | **0** | **1 (0.6)** |
| Pyrexia | 3 | 0 | 0 | 1 (0.6) |
| **Infections and infestations** | **3** | **1 (2.4)** | **4 (10.0)** | **10 (5.6)** |
| Lower respiratory tract infection | 3 | 0 | 1 (2.5) | 2 (1.1) |
| Bronchitis viral | 3 | 0 | 0 | 2 (1.1) |
| COVID-19 | 3 | 0 | 0 | 2 (1.1) |
| Upper respiratory tract infection | 3 | 0 | 0 | 2 (1.1) |
| Otitis media acute | 3 | 0 | 2 (5.0) | 0 |
| Gastroenteritis | 3 | 1 (2.4) | 0 | 1 (0.6) |
| Human herpesvirus 6 infection | 3 | 0 | 0 | 1 (0.6) |
| Nasopharyngitis | 3 | 0 | 0 | 1 (0.6) |
| Pneumonia | 3 | 0 | 0 | 1 (0.6) |
| Rotavirus infection | 3 | 0 | 0 | 1 (0.6) |
| Urinary tract infection | 3 | 0 | 0 | 1 (0.6) |
| Bone abscess | 3 | 0 | 1 (2.5) | 0 |
| Ear infection | 3 | 0 | 1 (2.5) | 0 |
| Gastrointestinal infection | 3 | 0 | 1 (2.5) | 0 |
| Mastoiditis | 3 | 0 | 1 (2.5) | 0 |
| **Vascular disorders** | **3** | **0** | **0** | **1 (0.6)** |
| Cyanosis | 3 | 0 | 0 | 1 (0.6) |

Prior to the second season, children with CHD/CLD randomized to nirsevimab in the first RSV season received 200 mg nirsevimab followed by four once-monthly doses of placebo (N/N) and those randomized to palivizumab in the first season were re-randomized 1:1 to either 200 mg nirsevimab followed by four once-monthly doses of placebo (P/N) or five once-monthly intramuscular doses of palivizumab (15 mg per kilogram of body weight per dose) (P/P). Adverse events were coded by MedDRA Version 23.1 or higher, unless otherwise stated.

^a^Relative to the active nirsevimab dose for P/N and N/N groups and to the first of 5 active doses for the P/P group.

^b^Grade 1 = Mild, Grade 2 = Moderate, Grade 3 = Severe, Grade 4 = Life-threatening, Grade 5 = Fatal.

^c^Cardiac surgery for Tetralogy of Fallot.

AT, as treated; CHD, congenital heart disease; CLD, chronic lung disease; COVID-19, coronavirus disease 2019; MedDRA, Medical Dictionary for Regulatory Activities; N, nirsevimab; P, palivizumab.

**Supplemental Table 2.** Overall Summary of Treatment-Emergent Serious Adverse Events through ≥150 Days Post First Dose^a^ of RSV Season 2 (AT population)

| Serious adverse events^b^ | P/P, *n* (%)  (*N* = 42) | P/N, *n* (%)  (*N* = 40) | N/N, *n* (%)  (*N* = 180) |
| --- | --- | --- | --- |
| **1 or more** | **0** | **4 (10.0)** | **17 (9.4)** |
| **Cardiac disorders** | **0** | **0** | **1 (0.6)** |
| Arrhythmia | 0 | 0 | 1 (0.6) |
| **Congenital, familial, and genetic disorders** | **0** | **0** | **1 (0.6)** |
| Fallot’s tetralogy | 0 | 0 | 1 (0.6)^c^ |
| **Gastrointestinal disorders** | **0** | **0** | **2 (1.1)** |
| Duodenal ulcer | 0 | 0 | 1 (0.6) |
| Intestinal obstruction | 0 | 0 | 1 (0.6) |
| **Infections and infestations** | **0** | **4 (10.0)** | **13 (7.2)** |
| Bronchitis viral | 0 | 0 | 3 (1.7) |
| Lower respiratory tract infection | 0 | 1 (2.5) | 2 (1.1) |
| COVID-19 | 0 | 0 | 2 (1.1) |
| Gastroenteritis | 0 | 0 | 2 (1.1) |
| Upper respiratory tract infection | 0 | 0 | 2 (1.1) |
| Pharyngitis | 0 | 0 | 1 (0.6) |
| Pneumonia | 0 | 0 | 1 (0.6) |
| Rotavirus infection | 0 | 0 | 1 (0.6) |
| Viral upper respiratory tract infection | 0 | 0 | 1 (0.6) |
| Bone abscess | 0 | 1 (2.5) | 0 |
| Ear infection | 0 | 1 (2.5) | 0 |
| Gastrointestinal infection | 0 | 1 (2.5) | 0 |
| Mastoiditis | 0 | 1 (2.5) | 0 |
| Otitis media | 0 | 1 (2.5) | 0 |
| Otitis media acute | 0 | 1 (2.5) | 0 |
| **Metabolism and nutrition disorders** | **0** | **0** | **1 (0.6)** |
| Failure to thrive | 0 | 0 | 1 (0.6) |
| **Nervous system disorders** | **0** | **1 (2.5)** | **0** |
| Nystagmus | 0 | 1 (2.5) | 0 |
| **Renal and urinary disorders** | **0** | **0** | **1 (0.6)** |
| Calculus urinary | 0 | 0 | 1 (0.6) |
| **Vascular disorders** | **0** | **0** | **1 (0.6)** |
| Cyanosis | 0 | 0 | 1 (0.6) |

Prior to the second season, children with CHD/CLD randomized to nirsevimab in the first RSV season received 200 mg nirsevimab followed by four once-monthly doses of placebo (N/N) and those randomized to palivizumab in the first season were re-randomized 1:1 to either 200 mg nirsevimab followed by four once-monthly doses of placebo (P/N) or five once-monthly intramuscular doses of palivizumab (15 mg per kilogram of body weight per dose) (P/P). Adverse events were coded by MedDRA Version 23.1 or higher, unless otherwise stated.

^a^Relative to the active nirsevimab dose for P/N and N/N groups and to the first of 5 active doses for the P/P group.

^b^Participants with multiple events in the same category were counted once in that category; participants with events in >1 category were counted once in each of those categories.

^c^Cardiac surgery for Tetralogy of Fallot; Grade 4 severity

AT, as treated; CHD, congenital heart disease; CLD, chronic lung disease; COVID-19, coronavirus disease 2019; MedDRA, Medical Dictionary for Regulatory Activities; N, nirsevimab; P, palivizumab.

**Supplemental Table 3.** Treatment-Emergent Adverse Events and Adverse Events of Grade 3 or Greater Severity Through ≥150 Days Post RSV Season 2 Dose by Time Relative to Any Dose^a^ (AT population)

| Adverse events^b^ | P/P, *n* (%)  (*N* = 42) | P/N, *n* (%)  (*N* = 40) | N/N, *n* (%)  (*N* = 180) | Total, *n* (%)  (*N* = 262) |
| --- | --- | --- | --- | --- |
| **1 or more** | **29  (69.0)** | **29  (72.5)** | **126 (70.0)** | **184 (70.2)** |
| Occurring ≤1 day post any dose^a^ | 0 (0.0) | 1 (2.5) | 4 (2.2) | 5 (1.9) |
| Occurring ≤3 days post any dose^a^ | 5 (11.9) | 8 (20.0) | 22 (12.2) | 35 (13.4) |
| Occurring ≤7 days post any dose^a^ | 8 (19.0) | 14 (35.0) | 41 (22.8) | 63 (24.0) |
| Occurring ≤14 days post any dose^a^ | 18  (42.9) | 15  (37.5) | 76  (42.2) | 109 (41.6) |
| **1 or more Grade 3 or greater severity^c^** | **1 (2.4)** | **4 (10.0)** | **14 (7.8)** | **19 (7.3)** |
| Occurring ≤1 day post any dose^a^ | 0 (0.0) | 0 (0.0) | 0 (0.0) | 0 (0.0) |
| Occurring ≤3 days post any dose^a^ | 0 (0.0) | 0 (0.0) | 1 (0.6) | 1 (0.4) |
| Occurring ≤7 days post any dose^a^ | 0 (0.0) | 0 (0.0) | 2 (1.1) | 2 (0.8) |
| Occurring ≤14 days post any dose^a^ | 0 (0.0) | 2 (5.0) | 3 (1.7) | 5 (1.9) |

Prior to the second season, children with CHD/CLD randomized to nirsevimab in the first season received 200 mg nirsevimab followed by four once-monthly doses of placebo (N/N) and those randomized to palivizumab in the first season were re-randomized 1:1 to either 200 mg nirsevimab followed by four once-monthly doses of placebo (P/N) or five once-monthly intramuscular doses of palivizumab (15 mg per kilogram of body weight per dose) (P/P). Adverse events were coded by MedDRA Version 23.1 or higher, unless otherwise stated.

^a^Refers to any dose of study drug in RSV Season 2 (i.e. for N/N and P/N nirsevimab or placebo; for P/P any palivizumab dose).

^b^Participants with multiple events in the same category were counted once in that category; participants with events in >1 category were counted once in each of those categories.

^c^An adverse event of Grade 3 denotes a severe event, an adverse event of Grade 4 denotes a life-threatening event, and an adverse event of Grade 5 denotes a fatal event.

AT, as treated; CHD, congenital heart disease; CLD, chronic lung disease; MedDRA, Medical Dictionary for Regulatory Activities; N, nirsevimab; P, palivizumab; TEAE, treatment-emergent adverse events.

**Supplemental Table ~~4~~.** Treatment-Emergent Adverse Events of Grade 3 or Greater Severity by Time Relative to Any RSV Season 2 Dose^a^ (1, 3, 7 or 14 days; AT population)

| **Adverse events of Grade 3 or greater severity** | **Highest severity grade^b^** | **P/P, *n* (%)**  **(*N* = 42)** | **P/N, *n* (%)**  **(*N* = 40)** | | | **N/N, *n* (%)**  **(*N* = 180)** | | | **Total, *n* (%)**  **(*N* = 262)** |
| --- | --- | --- | --- | --- | --- | --- | --- | --- | --- |
|  |  |  | **Nirsevimab dose** | **Placebo dose** | **Any dose** | **Nirsevimab dose** | **Placebo dose** | **Any dose** |  |
| **Within 1 day of any dosing** | | | | | | | | | |
| 1 or more | 3 | 0 (0.0) | 0 (0.0) | 0 (0.0) | 0 (0.0) | 0 (0.0) | 0 (0.0) | 0 (0.0) | 0 (0.0) |
|  | 4 | 0 (0.0) | 0 (0.0) | 0 (0.0) | 0 (0.0) | 0 (0.0) | 0 (0.0) | 0 (0.0) | 0 (0.0) |
| **Within 3 days of any dosing** | | | | | | | | | |
| 1 or more | 3 | 0 (0.0) | 0 (0.0) | 0 (0.0) | 0 (0.0) | 0 (0.0) | 1 (0.6) | 1 (0.6) | 1 (0.4) |
|  | 4 | 0 (0.0) | 0 (0.0) | 0 (0.0) | 0 (0.0) | 0 (0.0) | 0 (0.0) | 0 (0.0) | 0 (0.0) |
| Human herpesvirus 6 infection | 3 | 0 (0.0) | 0 (0.0) | 0 (0.0) | 0 (0.0) | 0 (0.0) | 1 (0.6) | 1 (0.6) | 1 (0.4) |
| **Within 7 days of any dosing** | | | | | | | | | |
| 1 or more | 3 | 0 (0) | 0 (0.0) | 0 (0.0) | 0 (0.0) | 0 (0.0) | 1 (0.6) | 1 (0.6) | 1 (0.4) |
|  | 4 | 0 (0) | 0 (0.0) | 0 (0.0) | 0 (0.0) | 0 (0.0) | 1 (0.6) | 1 (0.6) | 1 (0.4) |
| Arrhythmia | 3 | 0 (0) | 0 (0.0) | 0 (0.0) | 0 (0.0) | 0 (0.0) | 1 (0.6) | 1 (0.6) | 1 (0.4) |
| Fallot’s tetralogy | 4 | 0 (0) | 0 (0.0) | 0 (0.0) | 0 (0.0) | 0 (0.0) | 1 (0.6) | 1 (0.6) | 1 (0.4) |
| Human herpesvirus 6 infection | 3 | 0 (0) | 0 (0.0) | 0 (0.0) | 0 (0.0) | 0 (0.0) | 1 (0.6) | 1 (0.6) | 1 (0.4) |
| **Within 14 days of any dosing** | | | | | | | | | |
| 1 or more | 3 | 0 (0.0) | 0 (0.0) | 2 (5.0) | 2 (5.0) | 0 (0.0) | 2 (1.1) | 2 (1.1) | 4 (1.5) |
|  | 4 | 0 (0.0) | 0 (0.0) | 0 (0.0) | 0 (0.0) | 0 (0.0) | 1 (0.6) | 1 (0.6) | 1 (0.4) |
| Arrythmia | 3 | 0 (0.0) | 0 (0.0) | 0 (0.0) | 0 (0.0) | 0 (0.0) | 1 (0.6) | 1 (0.6) | 1 (0.4) |
| Fallot’s tetralogy | 4 | 0 (0.0) | 0 (0.0) | 0 (0.0) | 0 (0.0) | 0 (0.0) | 1 (0.6) | 1 (0.6) | 1 (0.4) |
| Human herpesvirus 6 infection | 3 | 0 (0.0) | 0 (0.0) | 0 (0.0) | 0 (0.0) | 0 (0.0) | 1 (0.6) | 1 (0.6) | 1 (0.4) |
| Pyrexia | 3 | 0 (0.0) | 0 (0.0) | 0 (0.0) | 0 (0.0) | 0 (0.0) | 1 (0.6) | 1 (0.6) | 1 (0.4) |
| Ear infection | 3 | 0 (0.0) | 0 (0.0) | 1 (2.5) | 1 (2.5) | 0 (0.0) | 0 (0.0) | 0 (0.0) | 1 (0.4) |
| Otitis media acute | 3 | 0 (0.0) | 0 (0.0) | 1 (2.5) | 1 (2.5) | 0 (0.0) | 0 (0.0) | 0 (0.0) | 1 (0.4) |

Prior to the second season, children with CHD/CLD randomized to nirsevimab in the first season received 200 mg nirsevimab followed by four once-monthly doses of placebo (N/N) and those randomized to palivizumab in the first season were re-randomized 1:1 to either 200 mg nirsevimab followed by four once-monthly doses of placebo (P/N) or five once-monthly intramuscular doses of palivizumab (15 mg per kilogram of body weight per dose) (P/P). AEs were coded by MedDRA Version 23.1 or higher, unless otherwise stated.

^a^Refers to any dose of study drug in RSV Season 2 (i.e. for N/N and P/N nirsevimab or placebo; for P/P any palivizumab dose).

^b^Grade 1 = Mild, Grade 2 = Moderate, Grade 3 = Severe, Grade 4 = Life-threatening, Grade 5 = Fatal.

AT, as treated; CHD, congenital heart disease; CLD, chronic lung disease; MedDRA, Medical Dictionary for Regulatory Activities; N, nirsevimab; P, palivizumab.
